# Improved anti-leukemia activities of adoptively transferred T cells expressing bispecific T-cell engager in mice

**DOI:** 10.1038/bcj.2016.38

**Published:** 2016-06-03

**Authors:** X Liu, D M Barrett, S Jiang, C Fang, M Kalos, S A Grupp, C H June, Y Zhao

**Affiliations:** 1 Center for Cellular Immunotherapies, University of Pennsylvania Cancer Center, Philadelphia, PA, USA; 2 Division of Oncology, Department of Pathology, Children's Hospital of Philadelphia, Philadelphia, PA, USA; 3 Department of Pathology and Laboratory Medicine, Perelman School of Medicine, University of Pennsylvania, Philadelphia, PA, USA

## Abstract

Despite the impressive clinical efficacy of T cells engineered to express chimeric antigen receptors (CAR-Ts), the current applications of CAR-T cell therapy are limited by major treatment-related toxicity. Thus, safer yet effective alternative approaches must be developed. In this study, we compared CD19 bispecific T-cell engager (BiTE)-transferred T cells that had been transfected by RNA electroporation with CD19 CAR RNA-transferred T cells both *in vitro* and in an aggressive Nalm6 leukemia mouse model. BiTEs were secreted from the transferred T cells and enabled both the transferred and bystander T cells to specifically recognize CD19^+^ cell lines, with increased tumor killing ability, prolonged functional persistence, increased cytokine production and potent proliferation compared with the CAR-T cells. More interestingly, in comparison with CD3/CD28 bead-stimulated T cells, T cells that were expanded by a rapid T-cell expansion protocol (REP) showed enhanced anti-tumor activities for both CAR and BiTE RNA-electroporated T cells both *in vitro* and in a Nalm6 mouse model (*P*<0.01). Furthermore, the REP T cells with BiTE RNAs showed greater efficacy in the Nalm6 leukemia model compared with REP T cells with CAR RNA (*P*<0.05) and resulted in complete leukemia remission.

## Introduction

Adoptive cell transfer (ACT) using chimeric antigen receptor (CAR-T) cells has demonstrated an unprecedented anti-leukemic response leading to the sustained remission in recent clinical trials.^1, 2, 3^ However, the current ACT approach using lentivirally or retrovirally transduced CAR-T cells has limitations associated with the lack of control over their activation and expansion *in vivo*,^4^ which has resulted in acute cases of tumor lysis syndrome and fatal cytokine release syndrome, as well as complications caused by the persistent on-target activity of CAR-Ts, such as long-term B-cell aplasia. RNA CAR-Ts, which are generated by electroporation of a messenger RNA encoding the CAR into the T cells, are being tested in clinical trials for the treatment of metastatic mesothelioma and pancreatic carcinoma.^5^ Potent anti-leukemia activities have also been demonstrated in preclinical mouse models using RNA CAR-Ts.^6, 7^ Because of the self-decay of the anti-tumor activity of RNA CAR-Ts, multiple infusions of a controlled dose of RNA CAR-Ts may limit tumor lysis syndrome and cytokine release syndrome. Moreover, the use of RNA CAR T cells may avoid causing long-term B-cell aplasia. However, the transient transgene expression in the RNA CAR-Ts leads to the suboptimal treatment efficacy compared with lentivirally transduced CAR-Ts.^6, 8, 9^ Efforts to further improve the anti-tumor activities of the RNA CAR-Ts will break this bottleneck and advance this RNA-based ACT platform, rendering it as potent as viral vector-based therapy, but potentially more flexible and safer.

Bispecific T-cell engagers (BiTEs), which are constructed by fusing an anti-CD3 scFv to an anti-tumor antigen scFv, have been tested in clinical trials for cancer treatment.^10, 11, 12^ However, the development of BiTEs to treat cancer patients still faces enormous challenges. BiTEs need to penetrate deeply into the tumor tissues, where the tumor resident T cells may have already been tolerized or anergized in the tumor microenviroment. In addition, the therapeutic potential of exogenously administered BiTEs may be limited by their short half-lives and their dissociation from triggering receptors within a relatively short period of time. Thus, integrating BiTEs with adoptive T-cell transfer *in situ* may enhance the cancer treatment efficiency of both BiTEs and adoptive T-cell transfer.^13, 14^

In this study, we tested the anti-leukemia activities of CD19 BiTE (blinatumomab) RNA-electroporated T cells that were generated through CD3/CD28 Dynal Bead stimulation or a rapid T-cell expansion protocol (REP) and found that the REP T cells transferred with a CD19 BiTE nearly completely eradicated the leukemia cells in the mice and resulted in sustained survival. Therefore, a combination of T cells generated by REP and the RNA electroporation of a CD19 BiTE has the potential to cure CD19^+^ malignancies with controlled toxicities and without B-cell aplasia.

## Materials and methods

### Cell lines and primary human T-lymphocyte cultures

The Nalm6 (DSMZ, Braunschweig, Germany), Raji (American Type Culture Collection, Manassas, VA, USA) and K562 (American Type Culture Collection) cell lines were cultured per the providers' instructions. The CD19-expressing K562 cells and click beetle green (CBG)-expressing Nalm6 cells were generated as previously described.^7^ Primary lymphocytes from normal donors were provided by the University of Pennsylvania Human Immunology Core. The primary T lymphocytes were stimulated and expanded using two different methods. (1) CD3/CD28 Dynabeads (Life Technologies, Grand Island, NY, USA) were used as previously described.^6^ (2) The REP approach was performed as previously described.^15^ In brief, 1 × 10^6^ purified CD4 and CD8 T cells in a 1:1 ratio were added to 1 × 10^8^ irradiated allogeneic peripheral blood mononuclear cells in a T150 flask in a total volume of 150 ml of R/10 medium in the presence of 50 ng ml^−1^ OKT3. Interleukin-2 (IL-2) was added to the culture for a final concentration of 300 IU ml^−1^ at day 2. At day 5, 120 ml of the culture supernatant was replaced with fresh R/10 medium containing 300 IU ml^−1^ of IL-2. The T cells were split every other day beginning 7 days after stimulation until day 11. The expanded T cells were aliquoted and frozen for further use.

### Construction of the *in vitro* transcribed (IVT) RNA vectors and RNA *in vitro* transcription and electroporation

The *in vitro* transcription vectors for the CD19-BBZ and CD19-28Z CARs were constructed as previously described.^7^ The DNA encoding the blinatumomab BiTE was synthesized based on the published sequence data from patent US7575923 and subcloned into a pGEM.64A-based *in vitro* transcription vector.^16^ The *in vitro* transcription vector was linearized by digestion with the proper restriction enzyme, and the mMESSAGE mMACHINE T7 Ultra kit (Life Technologies) was used to generate the IVT RNA, according to the procedure provided with the kit. The frozen stimulated T cells were thawed and cultured in R/10 medium overnight before electroporation. Before electroporation, the T cells were washed three times with OPTI-MEM (Life Technologies) and resuspended in OPTI-MEM (Life Technologies) at a final concentration of 1–3 × 10^8^ cells per ml before electroporation. Subsequently, 0.1 ml of the T cells was mixed with the indicated IVT RNA and electroporated in a 2-mm cuvette (Harvard Apparatus BTX, Holliston, MA, USA) using an ECM830 Electro Square Wave Porator (Harvard Apparatus BTX).^8^

### Enzyme-linked immunosorbent assay

The T cells or target cells were washed and suspended in R/10 medium at 1 × 10^6^ cells per ml. Approximately 0.1 ml of each cell line was added to a well of a 96-well plate (Corning) and incubated at 37 °C for 18–20 h. The supernatant was collected and subjected to an enzyme-linked immunosorbent assay.

### CD107a assay

The cells were plated at an effector:target (E:T) cell ratio of 1:1 (10^5^ effectors:10^5^ targets) in 160 μl of R/10 medium in a 96-well plate. An anti-CD107a antibody was added and incubated with the cells at 37 °C for 1 h before Golgi Stop was added and incubated for an additional 2.5 h. The anti-CD8 and anti-CD3 antibodies were added and incubated at 37 °C for 30 min. After incubation, the samples were washed once and subjected to flow cytometry with a BD FACSCalibur (BD Biosciences, Franklin Lakes, NJ, USA). The data were analyzed with the FlowJo software (FlowJo LLC, Ashland, OR, USA).

### CFSE-based T-cell proliferation assay

The RNA electroporation, stimulation and flow cytometry analyses were performed as previously described.^17^ In brief, resting CD4 T cells were washed and suspended in phosphate-buffered saline at a concentration of 1 × 10^7^ cells per ml. Then, carboxyfluorescein succinimidyl ester (CFSE) was added to the T cells at a final concentration of 2 μm at 25 °C for 3.5 min. The labeling reaction was stopped by adding 10 volumes of 5% fetal bovine serum (in phosphate-buffered saline), and the cells were then washed and cultured in R/10 medium. After an overnight culture, the CFSE-labeled T cells were electroporated with the indicated RNA. Two to four hours after electroporation, the T cells were suspended in R/10 medium at a concentration of 1 × 10^6^ cells per ml. The K562, K562-CD19 or K562-CD86 cell lines were irradiated and suspended in R/10 medium at 1 × 10^6^ cells per ml. The cells were plated at an E:T of 1:1 (5 × 10^5^ effectors:5 × 10^5^ targets) in 1 ml of complete R/10 medium in a 48-well plate. The T cells were then counted and fed every 2 days beginning at day 3. The CFSE dilution was monitored by flow cytometry at the indicated time points.

### Flow cytotoxic T-lymphocyte assay

A slightly modified version of a 4-h flow cytometry cytotoxicity assay was performed as previously described.^8, 18^

### Mouse xenograft studies

These studies were performed as previously described, with certain modifications.^6^ In brief, 6–10-week-old NOD–SCID-γc^−/−^ mice were bred in house under an approved Institutional Animal Care and Use Committee protocol. Approximately 1 × 10^6^ Nalm6-CBG cells were injected into each mouse via the tail vein. The T cells were injected via the tail vein 5 days after the Nalm6-CBG cells were injected. Tumor growth was monitored by bioluminescence imaging as previously described.^6^

### Statistical tests

To compare survival among the groups of treated mice, the log-rank (Mantel–Cox) test was used to determine statistical significance. The leukemia burdens, as measured by the bioluminescence imaging of the different groups, were compared with the Mann–Whitney test. Student's *t*-test was performed to compare differences in T-cell proliferation, lytic activity and cytokine levels.

## Results

### Functional BiTEs were secreted from transferred T cells loaded with BiTE RNA and exerted specific tumor reactivity

**Figure 1:**
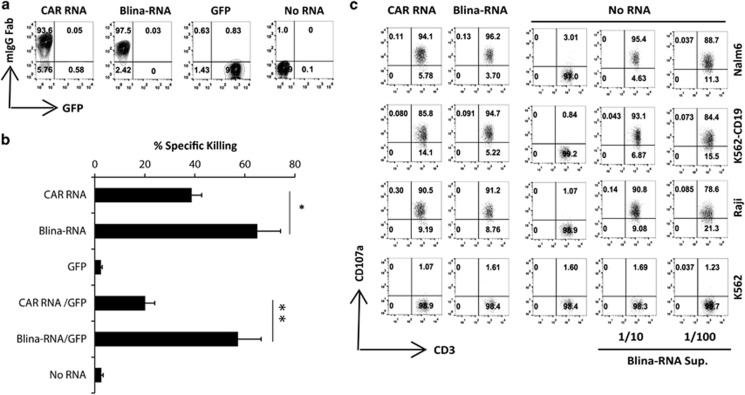
Blinatumomab BiTEs were secreted from RNA-transferred T cells and bound to T cells for tumor recognition. The T cells were electroporated with an RNA encoding CAR19 (CAR RNA), blinatumomab BiTEs (Blina-RNA) or GFP (at an RNA dose of 10 μg of RNA per 0.1 ml of T cells per electroporation). Eighteen hours after electroporation, the T cells were stained with a goat anti-mouse IgG Fab (mIgG Fab) to detect the expression of the CAR or blinatumomab on the T-cell surface (gated on CD3^+^ T cells) (**a**). Eighteen hours after electroporation, the CAR RNA or BiTE RNA T cells alone or mixed with an equal amount of GFP RNA T cells (GFP) were tested for their lytic activity using a cytotoxic T-lymphocyte assay at the effector:target ratio of 5:1 (**b**). The supernatant from the Blina-RNA T cells (Blina-RNA Sup.) was collected 18 h after electroporation, diluted 10 (1/10) or 100 times (1/100) with culture medium, added to T cells that were not electroporated with any RNA (No RNA) and co-cultured with the CD19^+^ cell lines (Nalm6, K562-CD19 or Raji cells). The K562 cell line was used as a negative control. T cells that had been electroporated with the CAR RNA or Blina-RNA were used as positive controls in the CD107a assay (gated on CD8^+^ T cells) (**c**) (representative of three independent experiments). *P<0.05; **P<0.01.

We performed experiments to investigate whether T cells transferred with a CD19 BiTE (blinatumomab) RNA (Blina-RNA) secreted functional BiTEs that were capable of engaging both the transfected and bystander T cells. Positive surface staining of the mIgG Fab of both CD19 CAR and blinatumomab was observed on the T cells that had been electroporated with either a FMC63 CD19-BBZ CAR RNA (CAR RNA) or the Blina-RNA (Figure 1a), suggesting that the BiTEs were secreted and loaded onto the surface of the T cells. A 4-h cytotoxic T-lymphocyte killing assay showed that Blina-RNA T cells killed the tumor cells more efficiently compared with the CAR RNA T cells (Figure 1b). When the CAR RNA or Blina-RNA T cells were mixed with an equal amount of green fluorescent protein (GFP) RNA-electroporated T cells, the CAR RNA T cells showed reduced lytic activity due to a decreased E:T ratio, whereas the killing ability of the Blina-RNA T cells remained as efficient as that of the non-diluted Blina-RNA T cells, presumably because the GFP RNA T cells were loaded with the blinatumomab that was secreted from the Blina-RNA-transferred T cells and thus maintained a higher E:T ratio. To further confirm that BiTEs were produced by the Blina-RNA T cells, the supernatant collected from the Blina-RNA-electroporated T cells was added to non-electroporated T cells in a CD107a assay. Indeed, the non-tumor-reactive T cells became highly tumor reactive when the supernatant collected from the BiTE RNA T cells was added (Figure 1c).

### The BiTE RNA T cells exhibited superior sensitivity and killing ability, with prolonged tumor reactivity

**Figure 2:**
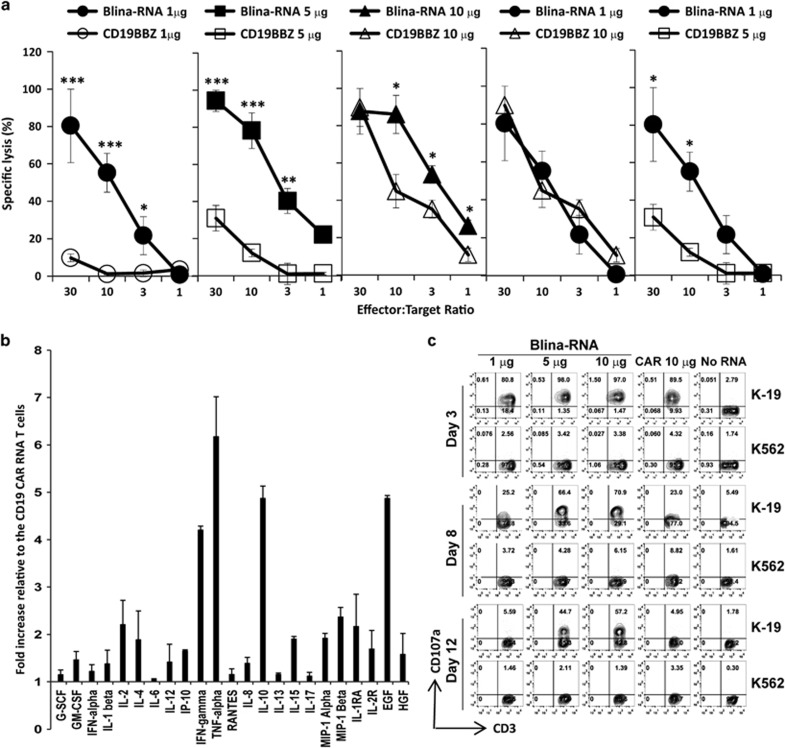
Blina-RNA T cells showed enhanced anti-tumor activities *in vitro*. T cells were electroporated with the blinatumomab RNA (Blina-RNA) or CD19-BBZ CAR RNA (at an RNA dose of 1, 5 or 10 μg per 0.1 ml of T cells per electroporation). Eighteen hours after electroporation, the lytic activity of the T cells against Nalm6 cells was tested (**a**). Eighteen hours after electroporation, 1 × 10^5^ T cells electroporated with 10 μg of either CD19-BBZ CAR RNA or Blina-RNA cells were stimulated overnight with 1 × 10^5^ CD19^+^ Nalm6 cells in a well of a 96-well plate. The supernatant was collected for the Luminex multiplex cytokine assay. The results showed the fold increase in the cytokine production of the Blina-RNA T cells relative to the CD19-BBZ CAR T cells (**b**). Blina-RNA T cells that had been electroporated with different amounts of the blinatumomab BiTE RNA (at 1, 5 and 10 μg per 0.1 ml of T cells) were compared with CAR RNA T cells that had been electroporated with 10 μg of the CD19-BBZ CAR RNA in a CD107a assay, and the results were plotted as dot plots of CD107a/CD3 expression at days 3, 8 and 12 (gated on CD8^+^ T cells) (**c**) (representative of two independent experiments). *P<0.05; **P<0.01; ***P<0.001.

It has previously been shown that T cells can be re-directed by loading an extremely low amount of BiTEs.^19^ Our previous work has shown that the T-cell activities of the RNA CAR-T cells are associated with the CAR RNA input.^6^ In a 4-h cytotoxic T-lymphocyte assay, the lytic ability of T cells transferred with 1, 5 or 10 μg of either the Blina-RNA or CD19-BBZ CAR RNA was compared. As shown in Figure 2a, the killing ability of the T cells correlated with the RNA dose. At the same RNA dose, the Blina-RNA T cells showed significantly stronger lytic activity compared with the CAR RNA T cells. T cells with 1 μg of the Blina-RNA killed the same number of tumor cells as T cells with 10 μg of the CAR19 RNA at different E:T ratios. However, there was no detectable lytic activity for the CAR RNA T cells with 1 μg of RNA, and the lytic activity of T cells with 5 μg of the CAR RNA was less effective than that of T cells with 1 μg of the Blina-RNA. In addition, Luminex multiplex cytokine analysis showed that the levels of most of the tested cytokines were increased, including both Th1-associated (interferon-gamma, tumor necrosis factor-alpha and IL-2) and Th2-associated (IL-4 and IL-10) cytokines, in the Blina-RNA T cells (Figure 2b) compared with the CAR RNA T cells. The antigen-specific T-cell activation at different times after electroporation was monitored by the upregulation of CD107a levels to analyze the T cells' functional persistence (Figure 2c). At day 3 post electroporation, the Blina-RNA T cells with different RNA doses (from 1 to 10 μg) showed anti-tumor activity as strong as that of the T cells with 10 μg of the CAR19 RNA. At day 8, the tumor reactivity of the T cells with 10 μg of the CAR19 RNA decreased to the level of the T cells with 1 μg of the Blina-RNA, whereas the tumor reactivity of the Blina-RNA T cells with 5 and 10 μg of RNA still remained high. Moreover, at day 12, a significant amount of CD107a remained expressed by T cells with 5 and 10 μg of the Blina-RNA.

### Reduced co-stimulatory dependence of BiTE RNA-transferred T cells

**Figure 3:**
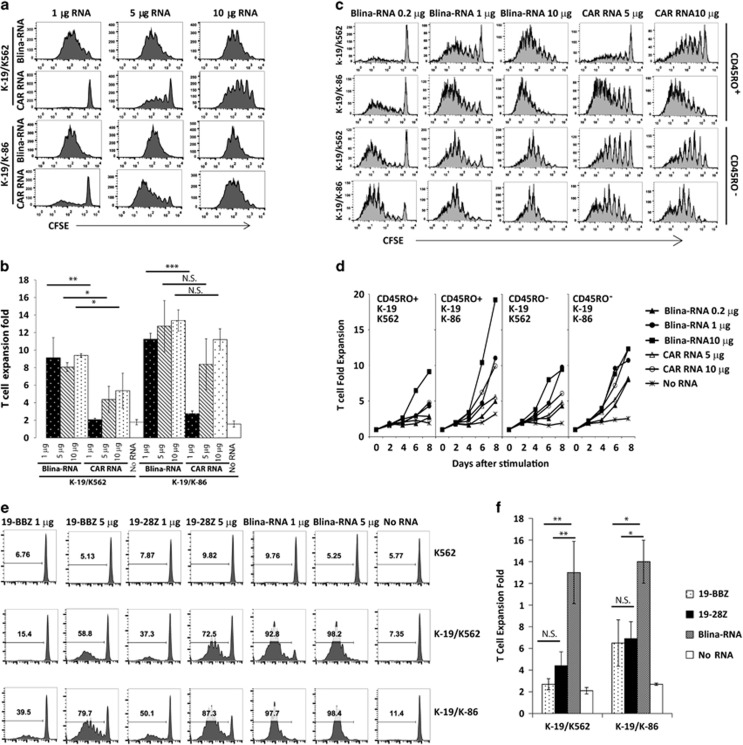
Blinatumomab BiTE RNA T cells were less dependent on co-stimulation and exhibited enhanced division and proliferation. CFSE-labeled resting CD4 T cells were electroporated with the blinatumomab BiTE RNA (Blina-RNA) or CD19-BBZ CAR RNA (CAR RNA) at the indicated RNA doses and stimulated with either irradiated K562-CD19 cells mixed with an equal amount of irradiated K562 cells (as control for K562-CD86; K-19/K562) or irradiated K562-CD19 cells mixed with an equal amount of irradiated K562-CD86 cells (K-19/K-86). The CFSE dilution was examined at day 6 (gated on CD3^+^ T cells). The results from a representative experiment are shown in **a**, and a summary of three independent experiments is shown in **b**. CFSE-labeled CD45RO^+^ (memory) or CD45RO^−^ (naive) resting CD4 T cells were electroporated with the blinatumomab BiTE RNA (Blina-RNA) or CD19-BBZ RNA (CAR RNA) at the indicated RNA doses and stimulated with either irradiated K562-CD19 cells mixed with an equal amount of irradiated K562 cells (as control for K562-CD86) or irradiated K562-CD19 cells mixed with an equal amount of irradiated K562-CD86 cells. The CFSE dilution was examined at day 6 (gated on CD3^+^ T cells) (**c**), and the T-cell expansion was monitored at different days after stimulation (**d**). The CSFE-labeled T cells that had been electroporated with either 1 or 5 μg of the CD19-BBZ (19BBZ), CD19-28Z (19-28Z) or blinatumomab BiTE (Blina-RNA) RNA were stimulated with irradiated K562, K562-CD19/K562 or K562-CD19/K562-CD86 cells. Six days later, the T cells were subjected to the flow cytometry analysis for CFSE dilution (gated on CD3^+^ T cells) (**e**). Five hundred thousand T cells that had been electroporated with 5 μg of the CD19-BBZ (19BBZ), CD19-28Z (19-28Z) or blinatumomab BiTE (Blina-RNA) RNA were stimulated with K562-CD19/K562 or K562-CD19/K562-CD86 cells. Six days later, the total number of viable T cells was counted, and the fold increase in T-cell expansion was calculated (**f**) (representative of two independent experiments). NS, not significant. *P<0.05; **P<0.01; ***P<0.001.

T-cell proliferation was evaluated by stimulating the T cells with CD19-expressing K562 (K562-CD19) cells with or without CD86-expressing K562 cells (K562-CD86). As shown in Figures 3a and b, when the T cells were stimulated with K562-CD19 cells and K562 cells without CD86 expression, the Blina-RNA T cells proliferated efficiently (Figure 3a) and exhibited 9.13±2.29-, 8.07±0.49- and 9.41±0.20-fold expansion for 1, 5 and 10 μg, respectively (Figure 3b). In contrast, the proliferation of the RNA CAR T cells was much less efficient, as evidenced by the nearly undetectable T-cell proliferation at an RNA dose of 1 μg. Even at an RNA dose of 10 μg, the proliferation of CAR RNA T cells was significantly less than that of Blina-RNA T cells. When CD28 co-stimulation was provided by stimulating the T cells with K562-CD19 and K562-CD86 cells, enhanced T-cell proliferation was observed for both the Blina-RNA and CAR RNA T cells, particularly the CAR RNA T cells, whereas CD28 co-stimulation only slightly increased the proliferation of Blina-RNA T cells, suggesting that the proliferation of the RNA CAR T cells was more dependent on co-stimulation. To further test whether the T cells could be efficiently activated with less Blina-RNA and whether the T-cell differentiation status influenced the stimulation, CFSE dilutions and the proliferation of CD45RO^+^ memory or CD45RO^−^ naive T cells were tested. The results showed that the T cells transferred with as low as 0.2 μg of Blina-RNA were efficiently stimulated to proliferate, particularly the CD45RO^−^ naive T cells. Moreover, when CD28 co-stimulation was provided, as evidenced by the CFSE dilution of T cells and cell expansion, the proliferation of 0.2 μg of Blina-RNA CD45RO^-^ T cells was generally equivalent to that of T cells with 5 μg of the CAR19 RNA (Figures 3c and d). The finding that CD45RO^−^ naive T cells with a low dose of Blina-RNA were more sensitive to the stimulation with or without CD28 co-stimulation is consistent with a recent finding that naive T cells proliferate at a lower threshold with reduced co-stimulation requirements compared with memory T cells.^20^

To exclude the possibility that this CD28 co-stimulation-dependent proliferation of CAR RNA T cells was due to the use of the BBZ configuration in the CAR construct, in which there is no CD28 signaling moiety, T cells transferred with the CD19-28Z CAR were used in a new experiment to assess the division and proliferation of CAR RNA- and Blina-RNA-electroporated T cells. There was no significant difference between the CD19-28Z and CD19-BBZ RNA T cells for both the CFSE dilution (Figure 3e) and T-cell expansion (Figure 3f) after stimulation with the K562-CD19 cells, with or without additional K562-CD86 cells. However, as shown above, the proliferation of both the CD19-28Z and CD19-BBZ RNA T cells was significantly lower than that of the Blina-RNA T cells, even in the presence of additional CD28 co-stimulation.

### T cells expanded by REP showed enhanced anti-tumor activities *in vitro*

**Figure 4:**
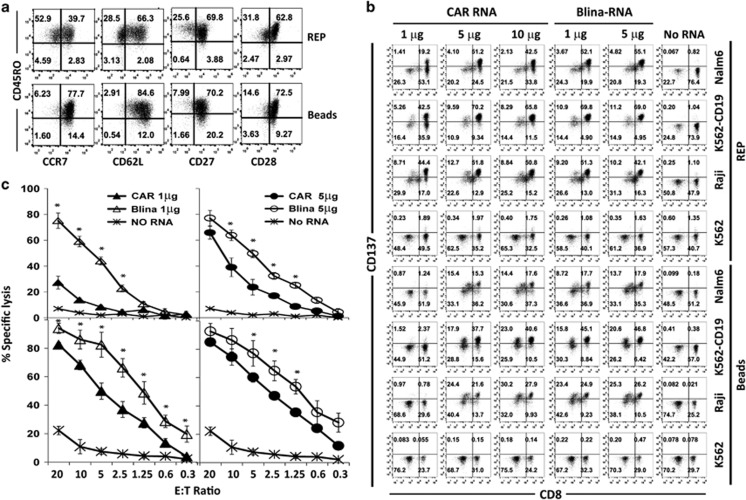
REP-expanded T cells improved the *in vitro* anti-leukemia activities of the blinatumomab BiTE RNA T cells. The phenotypes of the T cells that had been expanded by the REP or CD3/CD28 bead method (beads) are shown in (gated on CD3^+^ T cells) **a**. The REP T cells or CD3/CD28 bead-expanded T cells that had been electroporated with the indicated amounts of the CD19-BBZ CAR RNA or blinatumomab BiTE RNA were stimulated with the different cell lines for 18 h, and CD137 upregulation was detected by flow cytometry (gated on CD3^+^ T cells) (**b**). The lytic activity was measured for CD3/CD28 bead-expanded cells T cells (**c**, upper panel) or REP T cells (**c**, lower panel) that had been electroporated with different amounts of the CD19-BBZ CAR RNA (CAR) or blinatumomab BiTE RNA (Blina; representative of three independent experiments). *P<0.05.

Broad *ex vivo* cell expansion is required to produce a large number of effector T cells for adoptive immunotherapy, such as RNA electroporation-based adoptive immunotherapy. The REP approach expanded T cells up to 1000-fold in 2 weeks, which is ~10 times more than produced by CD3/CD28 bead stimulation (data not shown). Compared with T cells expanded by CD3/CD28 bead stimulation, which were primarily central memory T cells, the phenotypes of the REP-expanded T cells were more heterogeneous (CD45RO^+^/CCR7^+^ 32.00±9.07% (REP) versus 70±6.72% (beads), *P*<0.01), with fewer cells expressing CD62L (65.83±3.38% versus 85.02±11.13%, respectively, *P*<0.05), whereas there was no significant difference in CD27 (75.36±4.08% versus 80.97±18.73%, respectively, *P*>0.05) and CD28 expression (71.74±10.70% versus 78.92%±9.72%, respectively, *P*>0.05; Figure 4a). Unlike the REP-expanded tumor-infiltrating lymphocytes (TILs), which are mainly CCR7-negative effector or effector memory cells, the REP T cells that were expanded from peripheral blood mononuclear cells were heterogeneous, and the majority of this population maintained an young phenotype (Figure 4a). Upon stimulating the CAR RNA- or Blina-RNA-electroporated T cells that were generated by REP or CD3/CD28 bead stimulation, we found that the REP-expanded T cells showed enhanced anti-tumor activities for both the RNA CAR and Blina-RNA T cells, as evidenced by increased CD137 expression (Figure 4b) and increased lytic ability (Figure 4c), particularly when the RNA input dose was limited. Moreover, the Blina-RNA T cells showed superior *in vitro* anti-tumor activities compared with the RNA CAR T cells for both the CD3/CD28 bead and REP expansion methods (Figures 4b and c).

### REP-expanded T cells exhibited improved anti-leukemia activities, and sustained leukemia control was achieved using Blina-RNA-transferred REP T cells

**Figure 5:**
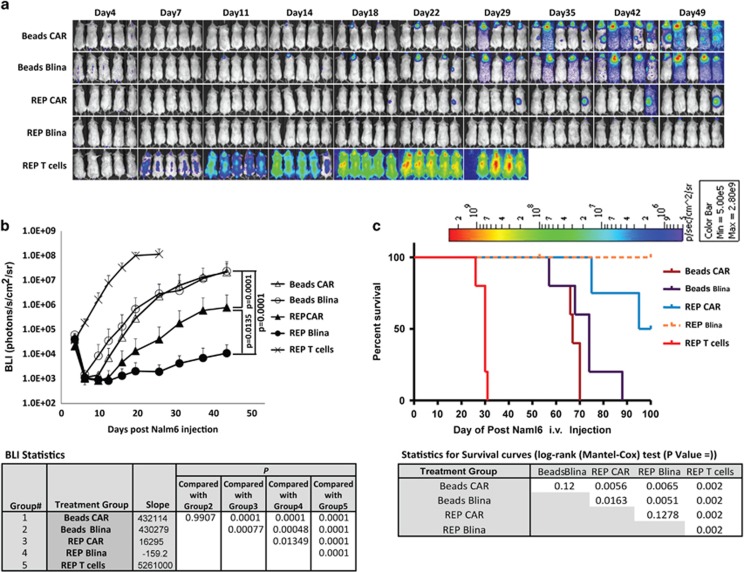
REP-expanded T cells further improved the *in vivo* anti-leukemia activities of the blinatumomab BiTE RNA T cells. NOD–SCID-γc^−/−^ mice were injected with 1 × 10^6^ Nalm6-CBG cells (intravenous), and 5 days later, the animals were treated with 3 × 10^7^ CD19-BBZ CAR RNA or Blina-RNA T cells generated by CD3/CD28 Dynal beads (beads) or REP, respectively, for the first treatment, followed by 5 × 10^6^ cells each, twice per week for 2 weeks, starting 8 days after the Naml6-CBG injection. The bioluminescence imaging (BLI) was conducted at the indicated time points (**a**), and the BLI and survival curves were plotted (**b**, **c**). Representative of three independent experiments.

To test whether the improved anti-tumor activity of the REP T cells *in vitro* correlated with their *in vivo* activity in a Nalm6 leukemia mouse model, the leukemia-bearing mice were treated with multiple injections of T cells electroporated with either the CD19-BBZ CAR RNA or Blina-RNA. As shown in Figure 5a, CD3/CD28 bead-expanded T cells that had been transferred with the CAR RNA or Blina-RNA partially controlled leukemia at similar levels. However, better leukemia control was achieved using both the RNA CAR and Blina-RNA REP T cells compared with the bead-expanded T cells (REP RNA CAR T cells versus bead CAR T cells: *P*<0.01; REP Blina-RNA T cells versus bead Blina-RNA T cells: *P*<0.001). Furthermore, the REP Blina-RNA T cells further improved the treatment compared with the CAR RNA T cells (*P*<0.05; Figure 5b). Consistently with results of the leukemia burden measured by bioluminescence imaging, a significant survival benefit was observed for the mice that had been treated with the Blina-RNA-transferred REP T cells (Figure 5c).

## Discussion

It has been shown that BiTEs are highly cytotoxic against various cell lines, using unstimulated human peripheral blood mononuclear cells in the absence of co-signaling, and that BiTEs can mediate the serial killing of many target cells.^19, 21^ Although effective, BiTEs have a short half-life, necessitating continuous systemic infusions, which may be associated with toxicity and a lack of active biodistribution, and, similarly to conventional monoclonal antibodies, they do not self-amplify.^12, 22^ The feasibility of delivering BiTEs using lentivirally transduced human T lymphocytes,^13, 14^ human mesenchymal stem cells and other cells^23, 24^ has been reported and tested in xenograft animal models, and these results have shown that integrating BiTEs with adoptive T-cell transfer *in situ* may overcome some of the shortcomings of systemic BiTE infusions to enhance the cancer treatment efficiency. However, although these studies have demonstrated the feasibility of redirecting T-cell activity with BiTE gene transfer, the methods generally do not allow us to control T-cell activities to prevent unwanted side effects, such as tumor lysis syndrome and cytokine release syndrome. In clinical studies with permanent CAR-engineered T cells that target CD19, the patients remained disease-free and displayed persistent engineered T cells for >4 years post treatment, although they also showed ongoing B-cell aplasia due to the targeting of normal CD19-positive B cells. Thus, these results highlight the practical need to eventually ablate the engineered cells and reconstitute normal B cells.

Because of the self-decay of the anti-tumor activity of RNA CAR-T cells, multiple infusions of a controlled dose of RNA CAR-Ts may potentially limit tumor lysis syndrome and cytokine release syndrome, and provide the additional benefit of avoiding long-term B-cell aplasia. However, the anti-tumor efficacy is limited by the transiently expressed CAR on the RNA-electroporated T cells compared with lenti- or retrovirally CAR-transduced T cells.^7, 8, 25^ In this study, we found that treating Nalm6 leukemia with REP T cells that were transferred with a blinatumomab RNA significantly prolonged the survival of the treated mice, results comparable to our observations in the same mouse model treated with CD19 CAR lentivirally transduced T cells. Therefore, the combination of T cells generated by REP with the RNA electroporation of BiTE RNAs has the potential to cure CD19^+^ malignancies with controllable toxicities and without long-term B-cell aplasia. Thus, integrating BiTEs with RNA-electroporated T cells may provide a safer and effective therapy to target CD19-positive tumors.

Currently, ACT uses viral and non-viral integration techniques that usually target only a single TAA. The selective pressure of an antigen-specific immune response may lead to the outgrowth of cancer cells lacking the target antigen, leading to incomplete treatment and treatment relapse, a phenomenon recently documented in failed treatments with CD19-specific CAR T cells.^26^ Preclinical ACT studies in mice have suggested that bystander destruction of the tumor has an important role in controlling tumors,^27, 28, 29^ and epitope spreading has been proposed as a mechanism by which T-cell-mediated killing of a limited population of tumor cells can lead to the immunologic destruction of other tumor cells expressing unrelated antigens.^30, 31^ T lymphocytes are considered to be antigen-presenting cells and are actively being incorporated into cancer vaccines.^32, 33^ A clinical trial using T cells that had been electroporated with a CAR messenger RNA targeting mesothelin to treat two cancer patients with pancreatic cancer and mesothelioma has reported that the T cells exhibited anti-tumor activity, and the repeated infusions of the messenger RNA CAR T cells elicited an anti-tumor immune response in both patients, as revealed by the development of novel anti-tumor antigen antibodies.^5^ A recent study has reported that proximal contact between T cells and live target cells through BiTEs directly reactivated the patients' pre-existing T cells to react with other cancer antigens via epitope spreading, thus supporting the hypothesis that the BiTE brings the T-cell receptor close to the tumor cell and facilitates direct recognition of new peptide–major histocompatibility complex epitopes on the tumor.^34^ Thus, repeated infusions of BiTE RNA T cells may not only kill the cancer cells that express the target antigen but also serve as a vaccine to mount immunologic immune responses against other tumor cells that express unrelated antigens, thus potentially preventing relapse from the outgrowth of targeted antigen-negative cancer cells.

Generally, it is believed that the T-cell anti-tumor efficiency primarily depends on the differentiation status of the adoptively transferred T cells, wherein T-cell differentiation is inversely correlated with the *in vivo* anti-tumor effectiveness.^35, 36^ The use of REP to expand TILs yields T cells with a loss of expression of CD28 and CD27;^37, 38^ these expanded TILs are prone to apoptosis and hypo-responsive to re-stimulation with tumor antigens.^39^ Instead of starting with TILs, T cells isolated from peripheral blood mononuclear cells were used for REP expansion in our current study. In contrast to T cells stimulated with soluble anti-CD3 antibody (OKT3) and IL-2, which produce more effector memory-type T cells with a significantly reduced number of CD62L^+^, CD28^+^ and CD27^+^ T cells that manifest inferior *in vivo* anti-leukemia ability compared with cells generated with CD3/CD28 beads,^40^ the majority of the REP-expanded T cells included both central memory and effector memory T cells and maintained a young phenotype (CD28^+^, CD27^+^ and CD62L^+^). When transferred with a CD19 CAR RNA or the Blina-RNA, those T cells not only exhibited more potent anti-tumor activity *in vitro*, probably because of the presence of more effector memory T cells, but also showed improved anti-tumor activity in an aggressive leukemia model compared with the CD3/CD28 bead-expanded T cells, which exhibited a more uniform central memory phenotype. These results suggest that T-cell heterogeneity may be important in controlling tumors, at least in T cells that are transiently transferred with CAR or BiTE RNAs, for which long-term persistence is not required, and proper migration and the maximum tumor killing ability are more important.

In summary, our study showed that BiTEs can be produced and delivered by T cells transferred with messenger RNAs encoding BiTEs, which may overcome the limitations of using exogenously administered BiTEs and improve the current CAR T cell-based adoptive immunotherapies against leukemia. Moreover, these T cells may be ideal vehicles to deliver other cargo molecules to orchestrate the tumor microenvironment and further facilitate anti-tumor activities. T cells generated using different methods for adoptive immunotherapy may significantly alter the outcome of the treatment; therefore, the selection of an optimized T-cell expansion and culture system is critically important to achieve the maximum therapeutic efficacy of this form of treatment.

## References

[bib1] Porter DL, Levine BL, Kalos M, Bagg A, June CH. Chimeric antigen receptor-modified T cells in chronic lymphoid leukemia. N Engl J Med 2011; 365: 725–733. 21830940 10.1056/NEJMoa1103849 PMC3387277

[bib2] Brentjens RJ, Davila ML, Riviere I, Park J, Wang X, Cowell LG et al. CD19-targeted T cells rapidly induce molecular remissions in adults with chemotherapy-refractory acute lymphoblastic leukemia. Sci Transl Med 2013; 5: 177ra38. 10.1126/scitranslmed.3005930 PMC3742551 23515080

[bib3] Lee DW, Kochenderfer JN, Stetler-Stevenson M, Cui YK, Delbrook C, Feldman SA et al. T cells expressing CD19 chimeric antigen receptors for acute lymphoblastic leukaemia in children and young adults: a phase 1 dose-escalation trial. Lancet 2015; 385: 517–528. 25319501 10.1016/S0140-6736(14)61403-3 PMC7065359

[bib4] Uttenthal BJ, Chua I, Morris EC, Stauss HJ. Challenges in T cell receptor gene therapy. J Gene Med 2012; 14: 386–399. 22610778 10.1002/jgm.2637

[bib5] Beatty GL, Haas AR, Maus MV, Torigian DA, Soulen MC, Plesa G et al. Mesothelin-specific chimeric antigen receptor mRNA-engineered T cells induce anti-tumor activity in solid malignancies. Cancer Immunol Res 2014; 2: 112–120. 24579088 10.1158/2326-6066.CIR-13-0170 PMC3932715

[bib6] Barrett DM, Zhao Y, Liu X, Jiang S, Carpenito C, Kalos M et al. Treatment of advanced leukemia in mice with mRNA engineered T cells. Hum Gene Ther 2011; 22: 1575–1586. 21838572 10.1089/hum.2011.070 PMC3237694

[bib7] Barrett DM, Liu X, Jiang S, June CH, Grupp SA, Zhao Y. Regimen-specific effects of RNA-modified chimeric antigen receptor T cells in mice with advanced leukemia. Hum Gene Ther 2013; 24: 717–727. 23883116 10.1089/hum.2013.075 PMC3746289

[bib8] Zhao Y, Moon E, Carpenito C, Paulos CM, Liu X, Brennan AL et al. Multiple injections of electroporated autologous T cells expressing a chimeric antigen receptor mediate regression of human disseminated tumor. Cancer Res 2010; 70: 9053–9061. 20926399 10.1158/0008-5472.CAN-10-2880 PMC2982929

[bib9] Barrett DM, Singh N, Liu X, Jiang S, June CH, Grupp SA et al. Relation of clinical culture method to T-cell memory status and efficacy in xenograft models of adoptive immunotherapy. Cytotherapy 2014; 16: 619–630. 24439255 10.1016/j.jcyt.2013.10.013 PMC3988256

[bib10] Bargou R, Leo E, Zugmaier G, Klinger M, Goebeler M, Knop S et al. Tumor regression in cancer patients by very low doses of a T cell-engaging antibody. Science 2008; 321: 974–977. 18703743 10.1126/science.1158545

[bib11] Klinger M, Brandl C, Zugmaier G, Hijazi Y, Bargou RC, Topp MS et al. Immunopharmacologic response of patients with B-lineage acute lymphoblastic leukemia to continuous infusion of T cell-engaging CD19/CD3-bispecific BiTE antibody blinatumomab. Blood 2012; 119: 6226–6233. 22592608 10.1182/blood-2012-01-400515

[bib12] Frankel SR, Baeuerle PA. Targeting T cells to tumor cells using bispecific antibodies. Curr Opin Chem Biol 2013; 17: 385–392. 23623807 10.1016/j.cbpa.2013.03.029

[bib13] Iwahori K, Kakarla S, Velasquez MP, Yu F, Yi Z, Gerken C et al. Engager T cells: a new class of antigen-specific T cells that redirect bystander T cells. Mol Ther 2015; 23: 171–178. 25142939 10.1038/mt.2014.156 PMC4426792

[bib14] Compte M, Blanco B, Serrano F, Cuesta AM, Sanz L, Bernad A et al. Inhibition of tumor growth *in vivo* by *in situ* secretion of bispecific anti-CEA x anti-CD3 diabodies from lentivirally transduced human lymphocytes. Cancer Gene Ther 2007; 14: 380–388. 17218946 10.1038/sj.cgt.7701021

[bib15] Dudley ME, Wunderlich JR, Shelton TE, Even J, Rosenberg SA. Generation of tumor-infiltrating lymphocyte cultures for use in adoptive transfer therapy for melanoma patients. J Immunother 2003; 26: 332–342. 12843795 10.1097/00002371-200307000-00005 PMC2305721

[bib16] Zhao Y, Boczkowski D, Nair SK, Gilboa E. Inhibition of invariant chain expression in dendritic cells presenting endogenous antigens stimulates CD4+ T-cell responses and tumor immunity. Blood 2003; 102: 4137–4142. 12920018 10.1182/blood-2003-06-1867

[bib17] Liu X, Jiang S, Fang C, Yang S, Olalere D, Pequignot EC et al. Affinity-tuned ErbB2 or EGFR chimeric antigen receptor T cells exhibit an increased therapeutic index against tumors in mice. Cancer Res 2015; 75: 3596–3607. 26330166 10.1158/0008-5472.CAN-15-0159 PMC4560113

[bib18] Hermans IF, Silk JD, Yang J, Palmowski MJ, Gileadi U, McCarthy C et al. The VITAL assay: a versatile fluorometric technique for assessing CTL- and NKT-mediated cytotoxicity against multiple targets *in vitro* and *in vivo*. J Immunol Methods 2004; 285: 25–40. 14871532 10.1016/j.jim.2003.10.017

[bib19] Mack M, Riethmuller G, Kufer P. A small bispecific antibody construct expressed as a functional single-chain molecule with high tumor cell cytotoxicity. Proc Natl Acad Sci USA 1995; 92: 7021–7025. 7624362 10.1073/pnas.92.15.7021 PMC41463

[bib20] Mehlhop-Williams ER, Bevan MJ. Memory CD8+ T cells exhibit increased antigen threshold requirements for recall proliferation. J Exp Med 2014; 211: 345–356. 24493801 10.1084/jem.20131271 PMC3920562

[bib21] Hoffmann P, Hofmeister R, Brischwein K, Brandl C, Crommer S, Bargou R et al. Serial killing of tumor cells by cytotoxic T cells redirected with a CD19-/CD3-bispecific single-chain antibody construct. Int J Cancer 2005; 115: 98–104. 15688411 10.1002/ijc.20908

[bib22] Fournier P, Schirrmacher V. Bispecific antibodies and trispecific immunocytokines for targeting the immune system against cancer: preparing for the future. BioDrugs 2013; 27: 35–53. 23329400 10.1007/s40259-012-0008-z

[bib23] Compte M, Cuesta AM, Sanchez-Martin D, Alonso-Camino V, Vicario JL, Sanz L et al. Tumor immunotherapy using gene-modified human mesenchymal stem cells loaded into synthetic extracellular matrix scaffolds. Stem Cells 2009; 27: 753–760. 19096041 10.1634/stemcells.2008-0831 PMC2729675

[bib24] Blanco B, Holliger P, Vile RG, Alvarez-Vallina L. Induction of human T lymphocyte cytotoxicity and inhibition of tumor growth by tumor-specific diabody-based molecules secreted from gene-modified bystander cells. J Immunol 2003; 171: 1070–1077. 12847281 10.4049/jimmunol.171.2.1070

[bib25] Singh N, Liu X, Hulitt J, Jiang S, June CH, Grupp SA et al. Nature of tumor control by permanently and transiently modified GD2 chimeric antigen receptor T cells in xenograft models of neuroblastoma. Cancer Immunol Res 2014; 2: 1059–1070. 25104548 10.1158/2326-6066.CIR-14-0051 PMC5584373

[bib26] Sotillo E, Barrett DM, Black KL, Bagashev A, Oldridge D, Wu G et al. Convergence of Acquired Mutations and Alternative Splicing of CD19 Enables Resistance to CART-19 Immunotherapy. Cancer Discov 2015; 5: 1282–1295. 26516065 10.1158/2159-8290.CD-15-1020 PMC4670800

[bib27] Spiotto MT, Rowley DA, Schreiber H. Bystander elimination of antigen loss variants in established tumors. Nat Med 2004; 10: 294–298. 14981514 10.1038/nm999

[bib28] Breart B, Lemaitre F, Celli S, Bousso P. Two-photon imaging of intratumoral CD8+ T cell cytotoxic activity during adoptive T cell therapy in mice. J Clin Invest 2008; 118: 1390–1397. 18357341 10.1172/JCI34388 PMC2268880

[bib29] Kerkar SP, Goldszmid RS, Muranski P, Chinnasamy D, Yu Z, Reger RN et al. IL-12 triggers a programmatic change in dysfunctional myeloid-derived cells within mouse tumors. J Clin Invest 2011; 121: 4746–4757. 22056381 10.1172/JCI58814 PMC3226001

[bib30] Hunder NN, Wallen H, Cao J, Hendricks DW, Reilly JZ, Rodmyre R et al. Treatment of metastatic melanoma with autologous CD4+ T cells against NY-ESO-1. N Engl J Med 2008; 358: 2698–2703. 18565862 10.1056/NEJMoa0800251 PMC3277288

[bib31] Bollard CM, Gottschalk S, Torrano V, Diouf O, Ku S, Hazrat Y et al. Sustained complete responses in patients with lymphoma receiving autologous cytotoxic T lymphocytes targeting Epstein-Barr virus latent membrane proteins. J Clin Oncol 2014; 32: 798–808. 24344220 10.1200/JCO.2013.51.5304 PMC3940538

[bib32] Fontana R, Bregni M, Cipponi A, Raccosta L, Rainelli C, Maggioni D et al. Peripheral blood lymphocytes genetically modified to express the self/tumor antigen MAGE-A3 induce antitumor immune responses in cancer patients. Blood 2009; 113: 1651–1660. 19074732 10.1182/blood-2008-07-168666

[bib33] Russo V, Cipponi A, Raccosta L, Rainelli C, Fontana R, Maggioni D et al. Lymphocytes genetically modified to express tumor antigens target DCs *in vivo* and induce antitumor immunity. J Clin Invest 2007; 117: 3087–3096. 17885685 10.1172/JCI30605 PMC1978420

[bib34] Dao T, Pankov D, Scott A, Korontsvit T, Zakhaleva V, Xu Y et al. Therapeutic bispecific T-cell engager antibody targeting the intracellular oncoprotein WT1. Nat Biotechnol 2015; 33: 1079–1086. 26389576 10.1038/nbt.3349 PMC4600043

[bib35] Gattinoni L, Klebanoff CA, Palmer DC, Wrzesinski C, Kerstann K, Yu Z et al. Acquisition of full effector function *in vitro* paradoxically impairs the *in vivo* antitumor efficacy of adoptively transferred CD8+ T cells. J Clin Invest 2005; 115: 1616–1626. 15931392 10.1172/JCI24480 PMC1137001

[bib36] Besser MJ, Shapira-Frommer R, Treves AJ, Zippel D, Itzhaki O, Hershkovitz L et al. Clinical responses in a phase II study using adoptive transfer of short-term cultured tumor infiltration lymphocytes in metastatic melanoma patients. Clin Cancer Res 2010; 16: 2646–2655. 20406835 10.1158/1078-0432.CCR-10-0041

[bib37] Li Y, Liu S, Hernandez J, Vence L, Hwu P, Radvanyi L. MART-1-specific melanoma tumor-infiltrating lymphocytes maintaining CD28 expression have improved survival and expansion capability following antigenic restimulation *in vitro*. J Immunol 2010; 184: 452–465. 19949105 10.4049/jimmunol.0901101

[bib38] Powell DJ Jr, Dudley ME, Robbins PF, Rosenberg SA. Transition of late-stage effector T cells to CD27+ CD28+ tumor-reactive effector memory T cells in humans after adoptive cell transfer therapy. Blood 2005; 105: 241–250. 15345595 10.1182/blood-2004-06-2482 PMC2553211

[bib39] Hernandez-Chacon JA, Li Y, Wu RC, Bernatchez C, Wang Y, Weber JS et al. Costimulation through the CD137/4-1BB pathway protects human melanoma tumor-infiltrating lymphocytes from activation-induced cell death and enhances antitumor effector function. J Immunother 2011; 34: 236–250. 21389874 10.1097/CJI.0b013e318209e7ec PMC3063939

[bib40] Jayasekara PS, Barrett MO, Ball CB, Brown KA, Hammes E, Balasubramanian R et al. 4-Alkyloxyimino derivatives of uridine-5'-triphosphate: distal modification of potent agonists as a strategy for molecular probes of P2Y2, P2Y4, and P2Y6 receptors. J Med Chem 2014; 57: 3874–3883. 24712832 10.1021/jm500367e PMC4018175

